# Correlation between Periodontitis and Gastritis Induced by *Helicobacter pylori*: A Comprehensive Review

**DOI:** 10.3390/microorganisms12081579

**Published:** 2024-08-02

**Authors:** Martina Maurotto, Liliana Gavinha Costa, Maria Conceição Manso, Grace Anne Mosley, Juliana Campos Hasse Fernandes, Gustavo Vicentis Oliveira Fernandes, Filipe Castro

**Affiliations:** 1 Institute of Research, Innovation and Development (FP-I3ID), Faculty of Health Science, Fernando Pessoa University, 4249-004 Porto, Portugal; 2 RISE-Health, Fernando Pessoa University, 4249-004 Porto, Portugal; 3 Missouri School of Dentistry & Oral Health, A.T. Still University, St. Louis, MO 63104, USA; 4 Independent Researcher, St. Louis, MO 63104, USA; 5 Department of Periodontology, University of Seville, 41009 Seville, Spain

**Keywords:** periodontitis, periodontal disease, dental plaque, *Helicobacter pylori*, dyspepsia, bacteria

## Abstract

The goal of this comprehensive review was to verify if the prevalence of *Helicobacter pylori* (*Hp*) bacteria in patients with dyspepsia is higher in the oral cavity of periodontal or non-periodontal patients. The bibliographic search was conducted on scientific studies published in PubMed, Cochrane Library, SciELO, and BVS. The focus question was: “In patients with dyspepsia and periodontitis, is the prevalence of Hp bacteria in the oral cavity higher than in patients with only dyspepsia or without any disease?” The inclusion criteria were human studies in English, Portuguese, or Spanish languages, published between 2000 and 2022, that included patients over the age of 18 and aimed to evaluate the presence of *Hp* bacteria in the oral cavity and in the protective mucosal layer of the gastric lining of patients with the diseases (periodontitis and dyspepsia) or without disease; clinical trials, randomized controlled clinical trials, comparative studies, case-control studies, cross-sectional studies, and cohort studies. The methodological quality evaluation of the included articles was performed using the Joanna Briggs Institute tools. The final scores could be of “Low” quality (at least two “no” [red] or ≥ five “unclear” found), “Moderate” quality (one “no” [red] was found or up to four “unclear” criteria were met), or “High” quality (all green [yes] or at maximum two “unclear”). Of 155 potentially eligible articles, 10 were included in this comprehensive review after the application of the eligibility criteria. The selected studies were scrutinized regarding the relationship between *Hp* colonization in the oral cavity and stomach, its impact on severity and complications of gastric infection, as well as the effect of the presence of oral and gastric *Hp* on dental and systemic parameters. *Hp* can colonize periodontal pockets regardless of its presence in the stomach. There was a higher prevalence of oral biofilm in dyspeptic patients with periodontal disease, and worse control of bleeding and low oral hygiene was observed in periodontal compared to non-periodontal patients. For que quality assessment, the scientific studies included presented low to moderate methodological quality. Conclusions: It is possible to conclude that *Hp* is a bacterium that can colonize dental plaque independently of the stomach and vice versa; however, when both diseases are found, its presence may be more significant. Supra and subgingival dental plaque may be a reservoir of *Hp*, suggesting that patients with gastric infections are more likely to have *Hp* in the oral cavity. The results must be carefully analyzed due to the limitations present in this review.

## 1. Introduction

Periodontitis, which was popularly known in the past as pyorrhea, according to the 2017 World Workshop on the Classification of Periodontal and Peri-implant Diseases and Conditions, is classified as an oral pathology caused by an inflammatory response on the periodontal tissues (alveolar bone, cement, gingiva, and periodontal ligament). These phlogistic phenomena generate dysbiosis within microbial communities, resulting in a process of progressive destruction of the supporting tissues. Radiographic bone loss (RBL), clinical attachment loss (CAL), gingival recession (Rec), periodontal pocket formation, the presence of bleeding on probing (in response to tissue manipulation), and often tooth suppuration and/or mobility are some of the clinical signs of this disease, which can ultimately compromise stability and thus lead to tooth loss [1,2].

Periodontal diseases are the most common oral pathologies, affecting approximately 90% of the world’s population. Age, sex, ethnicity, and socioeconomic and social conditions may be associated with an increase in the incidence and a more severe manifestation of the disease [3]. The literature has shown that the accumulation of dental plaque is directly proportional to the severity and prevalence of periodontal disease. Pathogenic microorganisms, including *Aggregatibacter actinomycetemcomitans*, *Porphyromona gingivalis*, *Tanerella forsythia*, and *Campylobacter rectus*, have been associated with periodontitis in several microbiological and epidemiological studies [4]. The disease is also related to the onset or worsening of systemic conditions. The field of periodontal medicine has led to the scientific publication of studies that correlate this oral pathology with the risk of systemic conditions, including chronic kidney disease [5], adverse outcomes during pregnancy, cardiovascular, diabetes, and respiratory diseases, Alzheimer’s disease, and cancer. In summary, it has been suggested that the periodontal bacterial component and/or the pro-inflammatory cascade associated with periodontitis could contribute to the pathogenesis of systemic diseases [6].

Gastrointestinal diseases have recently been linked to periodontitis, but as Gulati et al. [7] reported, more research is needed on the subject. At the digestive system level, *Helicobacter pylori* (*Hp*), a gram-negative bacterium that affects up to 50% of the world’s population, with a higher prevalence in developing countries, is a recognized etiological factor of chronic or atrophic gastritis, peptic ulcer, gastric lymphoma, and gastric carcinoma. These complications are less frequent in children and adolescents compared to adults. Its transmission can happen through fecal-oral, gastric, oral, or sexual routes, and its diagnosis can be made with invasive or non-invasive methods. Among the non-invasive methods, *Hp* antigens can be detected in feces, and antibodies against *Hp* can be found in serum, urine, oral samples, and urea breath tests. Invasive tests, on the other hand, use a sample of the individual’s gastric tissue to detect the presence of the bacteria through rapid urease tests, bacterial culture, histopathology, polymerase chain reaction, or fluorescent hybridization in situ. Concordant results from at least two tests are required to confirm that it is a *Hp* infection [8].

*Hp* was first identified in dental plaque in 1989 [9], which was considered a possible reservoir of this bacterium, especially in periodontal patients. This is because the environment of the periodontal pocket is favorable for this micro-aerophilic bacterium. He was alerted to a possible relationship between periodontal disease and gastric infection of *Hp* [7]. Their eradication in the stomach is not always successful, leading to the risk of recurrence or the progression of these infectious phenomena [10]. Faced with this new window on a possible relationship between periodontal disease and gastric infection by *Hp*, which is not yet well understood, possible questions emerge, such as whether: (1) the dental plaque supra and subgingival is a reservoir for *Hp*, (2) periodontal disease contributes to the increased presence of this bacterium in the oral cavity, (3) there is a relationship between *Hp*-induced gastric infection and the positive presence of this bacterium in the biofilm of periodontal patients, (4) the periodontal condition increases the risk of gastric infection from *Hp*. Thus, in order to improve and clarify the knowledge on Periodontal Medicine (the correlation between oral and systemic diseases), the questions raised about this theme may permit a better understanding of a possible link between periodontal disease and systemic pathological conditions of high prevalence, such as *Hp*-induced gastric infection.

The objective of this review was to verify the presence of *Hp* in the oral cavity of patients with dyspepsia and periodontal diseases. The null hypothesis was that no difference was found in the quantitative presence of *Hp* between non-periodontal and periodontal patients. This analysis will permit the verification of a positive or negative relationship between the two diseases. The result of this study has great potential for public health, as periodontal disease is preventable and treatable, and is a great opportunity to prevent/improve the prognosis of various systemic pathological conditions associated with this oral disease.

## 2. Materials and Methods

This comprehensive review was conducted based on the PRISMA (Preferred Reporting Items for Systematic Reviews and Meta-Analyses) guidelines [11] to better organize its methodology. The focus question was: “In patients with dyspepsia and periodontitis, is the prevalence of *Hp* bacteria in the oral cavity higher than in patients with only dyspepsia or without any disease?”

### 2.1. Search Strategy

The bibliographic search was carried out using four electronic databases: PubMed/MEDLINE, Cochrane Library, SciELO, and Bvsalud. The search key terms with the relative Boolean markers defined were: “(Periodontal Disease[MeSH] OR Periodontitis[MeSH]) AND (Dental Plaque[MeSH] OR Biofilm) AND (Helicobacter pylori[MeSH]) AND (Dyspepsia OR Gastri* OR Ulcer), with modulation according to the database (Table 1), on the publication’s abstract and/or title. In addition, the research was restricted to the period 2000–2022 and English language articles.

### 2.2. Study Selection and Inclusion Criteria

Two investigators (M.M. and F.C.) independently followed the search and selection of studies, and a third author (A.A.) collaborated in case of disagreement regarding the selection of articles. The inclusion criteria were: (1) human studies, (2) over the age of 18, (3) clinical trials, randomized controlled clinical trials, comparative studies, case-control studies, cross-sectional studies, and cohort studies, (4) articles aiming to evaluate the presence of *Hp* bacteria in the oral cavity and in the protective mucosal layer of the gastric lining of patients with the diseases (periodontitis and dyspepsia) or without disease, (5) articles published between 2000 and 2022, and (6) publications in English, Spanish, or Portuguese languages. The exclusion criteria were: (1) studies involving animals or only in vitro part, (2) reviews, letters to the editor, and case reports, (3) lack of information about the comparison between patients with and without diseases, (4) studies that included patients with age lower than 18 years.

### 2.3. Data Extraction

All the information reported that matched this study’s goal was retrieved. Two authors (M.M. and F.C.) performed this step; in case of disagreement, a third author (A.A.) was consulted. Specifically, the author’s name, year of publication, country of the study, type of the study, sample size and age of the patients, study population, the method used to diagnose *Hp* infection in the oral cavity and in the stomach, outcomes, and interpretation were retrieved.

### 2.4. Quality Assessment and Risk of Bias

The methodological quality assessment of the studies included in this review was evaluated using the Joanna Briggs Institute (JBI) tool. This analysis aimed to determine the extent to which a study presents the possibility of bias in its design, conduction, and analysis. There are four possible answers for each question: “Yes”, “No”, “Uncertain”, and “Not applicable” [12]. If the primary study meets all the criteria described, it receives a “Yes” answer in the question evaluated. If the study did not develop/evaluate in the way described in the question or did not mention the item in analysis, it receives the answer “No”. If it is not clear how that topic was developed, the answer is “Uncertain”. Two investigators (M.M. and F.C.) performed this step, and a third researcher (GVOF) was consulted in case of questions or doubts.

Finally, if the question does not apply to what is being analyzed, the answer is “It does not apply” [13]. The JBI tool provides for the exclusion of studies whose overall appraisal was of “Low” methodological quality. It is important to clarify that in the guidelines for the application of this tool, there are no cut-off points for the defined interpretation because the authors inform that the team must make the criteria explicit and agree among themselves to determine whether a study has “Low” quality (at least two “no” [red] or ≥five “unclear” found), “Moderate” quality (one “no” [red] was found or up to four “unclear” criteria were met), or “High” quality (all green [yes] or at maximum two “unclear”); the importance attributed to each criterion may vary according to the design of the included studies and the nature of the review under development [14].

## 3. Results

**Figure 1:**
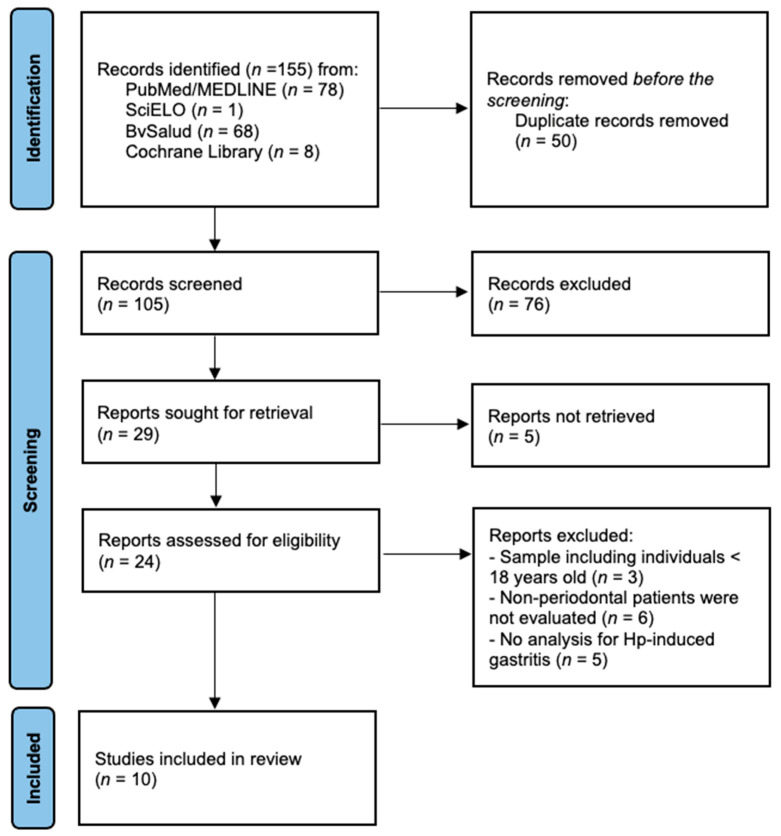
Flow diagram for the selection of the studies.

One hundred fifty-five articles were found, of which 50 were deleted because they were duplicates. After the deletion of these articles, an initial screening of the remaining articles was carried out by reading the title and abstract. Then, the other 81 articles were excluded, leaving 24 for full-text evaluation. Through the eligibility criteria previously stipulated for this review, 10 studies [9,10,15,16,17,18,19,20,21,22] were finally selected (Figure 1), which analyzed a total of 1685 patients.

The characteristics of the studies included in this comprehensive review and the results are presented in Table 2.

### 3.1. Relationship between Hp Colonization in the Oral Cavity and Stomach

Liu et al. [15] conducted a cross-sectional study with a population sample of 443 dyspeptic patients aged between 18 and 79. The aim was to evaluate the prevalence of gastric infection in dental plaque-positive *Hp* patients. The authors divided the sample into four groups of age: 18–29; 30–39; 40–49; ≥50. In all groups, a statistically higher prevalence of gastric infection was found in *Hp*+ patients in dental plaque compared to *Hp*− [15].

Alagl et al. [10], with the same design as the previous study, evaluated the association between gastric colonization and oral *Hp*. One of its objectives was to analyze the association between oral *Hp* colonization and dental diseases. Most of the patients included in the sample had good oral hygiene levels and moderate socioeconomic status. The results of the study did not show a significant association between the oral and gastric presence of PH and periodontal diseases in these patients. On the other hand, patients with *Hp*+ gastritis have been shown to have a higher number of decayed and missing teeth [10].

Eskandari et al. [17] used a sample of 67 patients with chronic periodontitis, 23 of whom also suffered from gastritis. The authors assessed the presence of *Hp* in dental plaque and determined whether an association with *Hp*+ gastritis was present. This study determined a positive association between the two variables (*p* = 0.012) [17].

Another study that addressed the association between the presence of *Hp* in dental plaque and the stomach of gastritis patients, and the effect of oral hygiene and periodontal condition on the correlation with gastritis, was conducted by Al-Refai et al. [10]. The sample comprised 75 Saudi adult dyspeptic patients and 60 healthy people as controls. With a statistically significant result (*p* < 0.0001), a high association was shown between plaque urease and gastric urease; otherwise, no association was observed between plaque and stomach ureases with oral hygiene parameters and dental, gingival, and periodontal plaque indexes used in the study [10].

Al Asqah et al. [19] conducted a cross-sectional study on 101 patients with dyspepsia to evaluate whether the presence of *Hp* in the dental plaque of periodontal and non-periodontal patients had a correlation with gastric pathology. The 101 patients were divided into two groups: (A) periodontal and (B) non-periodontal. Patients with periodontitis had a significantly higher percentage of *Hp* in dental plaque (79% versus 43%; *p* < 0.05) and stomach (60% versus 33%; *p* < 0.05) than in patients without periodontitis. Twenty-nine (78%) of 37 periodontal patients had *Hp*+ in their plaque samples; in the non-periodontal population of 13 patients, only four (30%) had the bacteria in the dental plaque, a statistically significant difference (*p* < 0.05) [19].

Silva Rossi-Aguiar et al. [20], in a cross-sectional study on 43 patients with epigastric pain syndrome, evaluated the presence of *Hp* in the oral cavity. *Hp* was found in the stomachs of 30 patients (69.7%). A total of 144 samples were collected from the oral cavity. Among them, 80 (55.5%) were positive for the rapid urease test, but the PCR technique did not detect *Hp* DNA in any oral sample [20].

Silva et al. [21] evaluated the presence of oral *Hp* in periodontal and gastric patients. The authors conducted a study on 115 patients subdivided into four groups: (A) with gastric diseases and periodontal disease; (B) with gastric diseases and without periodontal disease; (C) without gastric diseases and without periodontal disease; and (D) without gastric diseases and with periodontal disease [21]. *Hp* was detected in supragingival plaque (25%) of group A, 0.3% of group B, not detected in group C, and 8.3% in group D. No *Hp*+ positive subgingival samples were found. A statistically higher prevalence of *Hp* resulted in groups A and D when compared to groups B and C (*p* < 0.05).

Bago et al. [22] evaluated the presence of *Hp* in the oral cavity of patients with *Hp*+ gastric infection in a sample of 94 people. Of the 56 individuals with chronic periodontitis, *Hp* was detected in the oral cavity of 23 patients (41.1%). Twelve saliva samples (21.4%), 15 (26.8%) supragingival plaque samples, and 17 (30.4%) subgingival plaque samples were positive for *Hp*.

### 3.2. Impact of Oral Hp on the Severity and Complications of Gastric Infection

In the study by Ansari et al. [16], the authors studied 576 periodontal patients dividing the sample into two parts: a group of cases with infection or gastric complications of 278 people, and a control group of 289 patients. The aim of the study was to identify the presence of *Hp* in the oral mucosa and discover its relationship with infection and gastric complications. The results obtained were a negative association between the oral presence of the bacterium and gastric infections/complications between the group of cases and controls; but on the contrary, it was shown that the pathogenic genes of oral *Hp* can increase the severity of gastric infection, increasing the risk of developing conditions such as gastritis, gastro-oesophageal reflux, and the mucosa of the upper gastrointestinal tract normal with lax esophageal sphincters [16].

### 3.3. Effect of the Presence of Oral and Gastric Hp on Dental and Systemic Parameters

In Bürgers et al.’s (2008) article, the concomitant presence of *Hp* in the oral cavity and stomach and the impact of the presence of the bacterium on dental and systemic parameters were evaluated. In this study, only 6 out of 94 patients evidenced the concomitant presence of the bacterium in the oral cavity and in gastric biopsy samples, and most of the parameters analyzed, such as number of teeth, deeper periodontal pocket, body mass index, smoking habit, and age, did not show a statistically significant correlation [18].

### 3.4. Quality Assessment and Risk of Bias

The methodological quality of the studies included in this review (Table 3) was assessed using the Joanna Briggs Institute (JBI) tool. According to the nature and objectives of this study, a valid and reliable measurement of *Hp* at the oral and gastric levels is the essential parameter to assess its prevalence and respond to the objectives of this analysis.

Of the articles included in this review, for the definition of the inclusion criteria of the sample, two [10,18] presented a high risk of bias. For the description of the sample and its context, four presented a high quality [10,20,21,22]. In terms of outcome measurement, all of them had a low risk of bias. When confounding factors were analyzed, three achieved a high risk [16,20,22]. All of the results showed high methodological quality when measured. At the level of statistical analysis, only one [20] presented a high-risk element. In general, the scientific studies included in this review had low to moderate methodological quality.

## 4. Discussion

The goal of this comprehensive review was to verify the presence of *Hp* in the oral cavity of patients with concomitant dyspepsia and periodontal diseases. Both diseases have common risk factors (smoking, uncontrolled diabetes, poor socioeconomic status and health, and alcohol consumption), which means that the presence of one of these diseases may exacerbate the other [23]. In a systematic review with meta-analysis, Wei et al. [24] reported that *Hp* increased the odds ratio of chronic periodontal disease by 3.42 times; this result agrees with another meta-analysis outcome [25] that reported the presence of *Hp* infection increased the risk of periodontitis by 2.47 times. It was evident, and both meta-analysis articles concluded, that there exists an association between *Hp* infection and periodontitis. Thus, identifying the factors affecting the diseases can be useful for the development of appropriate and effective care strategies. It is important to highlight that the best long-term evidence suggests that toothbrushing twice or more a day is correlated with a lower-level periodontal pocket depths (≥4 mm) development over a period of 11 years [26]. In addition, when the quality of self-performed mechanical plaque removal is not completely effective, there is a higher risk of periodontal disease. There are no alternative methods currently more effective than mechanical control; therefore, chemical adjuncts in dentifrices and mouth rinses have provided additional benefits in the prevention of gingivitis. Otherwise, the complete biofilm removal through mechanical activities remains limited for various reasons: (1) incomplete biofilm removal due to limitations in the patient’s dexterity and precision and (2) oral hygiene devices allow, sometimes, limited access in specific cases of anatomic and morphologic changes (tooth crowding and root irregularities) [26]. However, a question remains: How much bacterial plaque needs to be removed to achieve the periodontal health condition using primary prevention has not been defined [27]. Regarding interdental cleaning, the use of an interdental brush has been considered the most effective way for periodontitis patients; therefore, the alternative use of dental floss, wood sticks, or rubber interdental cleaners in case the interdental brush does not appropriately fit without trauma [26] has also been reported.

The influence of the presence of oral *Hp* in patients affected by *Hp*+ gastric infection was demonstrated in the cross-sectional study by Liu et al. [15]. However, it is essential to report some limitations observed; the detection method used to assess the presence of *Hp* was the urease rapid test as a relatively specific and sensitive *Hp*+ gastric infection diagnostic test. This is an advantage because only this bacterium can produce urease in the stomach with high activity. In fact, the result produced by this type of test should be evaluated with caution because, in the oral cavity, there are many other bacteria that contain urease, which may cause false-positive results. It would be useful to do a molecular analysis of the DNA from oral and gastric strains to compare and have a more realistic outcome for this relationship [15].

Studies on this subject have been carried out [28,29]. In the first mentioned [28], the bacteria were cultured from the saliva of one of nine patients who tested positive for gastric *Hp*. The sampling was identical to that derived from the antral biopsy of the same patient. It differed from the cultures of all other patients in soluble protein electrophoresis, restriction endonuclease DNA analysis, and southern spot hybridization. Young et al. [29] conducted an endoscopic on five patients’ upper gastrointestinal and oral plaque samples; they also reported no morphological differences after scanning electron microscopy (SEM) analyses.

Another important question to report and analyze was whether colonization of this bacterium began at the oral level first and then reached the stomach or vice versa. Both options are viable; if it was assumed initial colonization of *Hp* in the oral cavity and then gastric, it is considered bacterial transmission by oral-oral or oral-fecal; on the other hand, if there was a primary gastric colonization, it is possible to assume that, through the mechanisms of gastro-esophageal reflux, the bacterium moved orally. Further studies are needed to better answer this question. In addition, there is evidence that a percentage of the population had the presence of oral *Hp*, but not *Hp*+ gastritis and vice versa. It is possible to assume that, in the first scenario, it happened because of a low absolute number of bacteria, not enough for gastric colonization; and, in the second, because the bacteria were not found within the oral cavity. Further studies would be needed to address these issues [15].

Opposite results were achieved by Alagl et al. [10], who showed that only a non-significant small fraction of the sample had *Hp* in their mouth and that there was no association with periodontal disease. Also, in this case, it is necessary to expose some limitations, especially at the population sample level and the bacterial quantification tests used. First, the study [10] analyzed a small population (120 patients), including mainly older women; it cannot be considered representative of a national population, which brought bias to the selection. Another essential issue to highlight is that most of these periodontal patients have a moderate socioeconomic level and good oral hygiene. Therefore, it is not possible to generalize these results broadly to periodontal patients because they have the disease at different stages and degrees, and not all carry out adequate bacterial control. However, it is also necessary to report that this study [10] obtained different results in terms of prevalence than those previously conducted, according to the authors; therefore, according to the literature, the prevalence of this microorganism varies in different populations. Another flaw present was in the measurement of the bacterium at the gastric sample level; as already previously exposed in this review, concordant results of at least two tests are needed to confirm a *Hp* infection [8]; otherwise, the authors of this research [10] used only Giesma staining for the detection of the microorganism.

The study by Ansari et al. [16] identified the *Hp*’s virulence factors cagA, vacA, and babA2 as responsible for the increased risk of gastric complications but did not show statistically significant differences between the presence of the bacterium in the oral cavity of periodontal patients with and without gastric complications. It is necessary to underline that a very mixed population was analyzed because the patients studied had great heterogeneity (moderate and severe gingivitis; and mild, moderate, and severe periodontitis).

In the study by Eskandari et al. [17], a significant association was demonstrated between the presence of oral *Hp* and gastritis (*p* = 0.012). Otherwise, the fact that the bacterium was found in only 5.9% of the periodontal patients enrolled represents a limitation for the generalization of the results obtained. The detection rate of this microorganism is very variable in the different scientific research studies (0–90%), mainly due to the methodological differences among them; one of the reasons given on the subject is because the literature is still very controversial. For example, in this publication [17], the authors referred to the use of the PCR test as the one with the highest *Hp* detection rate compared to the other less reliable and specific ones, such as the rapid urease test. However, the study with PCR depends a lot on the sensitivity and specificity of the primers used, which also justifies the difference between the results obtained with the same method in several studies.

Bürgers et al. [18] made an observation of the bacterium’s site in the oral cavity. They stated in their cross-sectional study that, although *Hp*’s DNA was more often found on the dorsum of the tongue and saliva than dental plaque, it cannot properly recommend preferable colonized sites. In this investigation [18], the authors reported that their results did not support a positive correlation between bacterial colonization of the stomach and the oral cavity and vice versa. The researchers also studied several dental and general health parameters analyzed for their influence on the presence of *Hp* in the oral cavity and stomach. Most parameters (number of teeth, deeper periodontal pocket, body mass index, smoking habit, and age) did not correlate statistically. It is important to report that women had a slightly higher percentage of oral *Hp*, and males had an increased prevalence of *Hp*+ infection in the stomach and serum positivity. To overcome the difficulty of recognizing the bacterium among a microbiota as vast as the oral microbiota, where there are more than 700 species, the authors used DNA sequencing after analysis with primers (PCR). Culture analysis is very unfeasible, probably due to the low presence of the bacterium in the oral cavity. The results of the assay did not support the presence of *Hp* in a normal oral microflora, an argument that has also not yet been clarified [18].

Al-Refai et al. [10] showed a highly statistically significant association between plaque urease and gastric urease (*p* < 0.0001), suggesting the ability to detect *Hp* in dental plaque samples with potential non-invasive gastric infection testing and support oral spread of *Hp* as the primary mode of transmission. The authors presented it as a target for therapy and a tool for monitoring the efficacy of eradication (the presence of the bacterium in dental plaque and the stomach [in gastric patients]). The fact that at least two gastric biopsy samples were analyzed by each patient to be evaluated gave more reliability to the results obtained.

In a cross-sectional study [19], the authors showed that patients with poor oral hygiene have a higher prevalence of *Hp* in the oral plaque and stomach, suggesting that the oral cavity may effectively be a reservoir or a source of potential transmission or reinfection. In a completely opposite conclusion, Silva Rossi-Aguiar et al. [20] stated that the oral cavity could not be a reservoir of *Hp* in patients with epigastric pain syndrome, and the bacterium was detected exclusively in the stomach. Therefore, this statement, as well as this study [20], have limitations. The PCR test used for the detection of *Hp* in the oral cavity may be due to the low sensitivity of the method due to a low number of microorganisms in the specimens, problems in DNA isolation, or the presence of inhibitors of PCR reactions [30].

Silva et al. [21] found *Hp* in the supragingival oral plaque but not in the subgingival area of individuals with periodontal and upper gastric diseases. An association was identified between supragingival *Hp* colonization and oral hygiene parameters, such as the presence of dental bacterial plaque and gingival bleeding. It is important to emphasize that the oral sample collection was carried out only in posterior teeth, casting doubt on the real possibility of representing the entire oral cavity. Another issue to be highlighted is the methodology and the instruments used to collect the oral sample. In this trial [21], sterile paper tips were used, but the use of periodontal curettes, in reality, by collecting dental plaque from subgingival pockets, provides more material for analysis than the paper tips, although contamination of the sample with supragingival material is almost inevitable.

Bago et al.’s study [22] was a cohort study that evaluated the presence of *Hp* in the oral cavity of patients with *Hp*+ gastric infection and examined the efficacy of eradication therapy against the bacteria in the stomach and oral cavity. It has been shown that almost half of the *Hp*+ patients in the stomach also had the bacteria orally.

As limitations of this study, it is possible to consider the low number of clinical studies analyzing and comparing *Hp* in both disease conditions; the low number of articles with a low sample size achieved and greater part with low to moderate quality, suggesting future studies to expand the search to years before than 2000; and the limited number of databases used for consultation (PubMed/MEDLINE, Cochrane Library, SciELO, and Bvsalud). The general moderate-to-low quality found in the studies, and the different tests used can be mentioned, which raised questions about efficacy and reliability.

## 5. Conclusions

Within the limitations of this comprehensive study, it is possible to conclude that *Hp* is a bacterium that can colonize dental plaque independently of the stomach and vice versa; however, when both diseases are found, its presence may be more significant. Then, supragingival or subgingival plaque may be considered a reservoir of *Hp* bacteria; patients with gastric infections are more likely to have *Hp* in the oral cavity, which seems to increase the occurrence of gastric infection complications.

Thus, based on the studies evaluated, it is possible to reject the null hypothesis. Therefore, the results must be carefully analyzed due to the limitations present in this review. The periodontal dyspeptic patient with poor plaque control and poor oral hygiene has a higher prevalence of *Hp* in the periodontal pocket compared to non-periodontal patients. However, there was a quantitative reduction when good oral hygiene conditions and moderate socioeconomic status were verified. Studies suggest that professional procedures for plaque removal and oral hygiene combined with antibiotic treatment of *Hp* make it possible to improve the eradication rate and decrease the risk of reinfection. In this sense, more studies are suggested under this theme, employing more standardized protocols in order to confirm the strong relationship between these two pathologies.

## Figures and Tables

**Table 1:** Bibliographic research strategy carried out.

Database	Terms	Query
PubMed/MEDLINE	periodontal disease or periodontitis and dental plaque and helicobacter pylori	(“periodontal diseases”[MeSH Terms] OR (“periodontal”[All Fields] AND “diseases”[All Fields]) OR “periodontal diseases”[All Fields] OR (“periodontal”[All Fields] AND “disease”[All Fields]) OR “periodontal disease”[All Fields] OR (“periodontal”[All Fields] OR “periodontally”[All Fields] OR “periodontically”[All Fields] OR “periodontics”[MeSH Terms] OR “periodontics”[All Fields] OR “periodontitis”[MeSH Terms] OR “periodontitis”[All Fields] OR “periodontit*”[All Fields] Fields])) AND (“dental plaque”[MeSH Terms] OR (“dental”[All Fields] AND “plaque”[All Fields]) OR “dental plaque”[All Fields]) AND (“*Helicobacter pylori*”[MeSH Terms] OR (“*Helicobacter*”[All Fields] AND “*pylori*”[All Fields]) OR “*Helicobacter pylori*”[All Fields])
SciELO	periodontal disease or periodontitis and dental plaque and helicobacter pylori	(((Periodontal Disease) OR (Periodontitis)) AND (Dental Plaque)) AND (*Helicobacter pylori*)
Cochcrane Library	periodontal disease or periodontitis and dental plaque and helicobacter pylori	(((Periodontal Disease) OR (Periodontitis)) AND (Dental Plaque)) AND (*Helicobacter pylori*)
Bvsalud	periodontal disease or periodontitis and dental plaque and helicobacter pylori	(((Periodontal Disease) OR (Periodontitis)) AND (Dental Plaque)) AND (*Helicobacter pylori*)

**Table 2:** Characteristics of the studies included and their results.

Author (Year)	Parents	Study Design	Sample (Age)	Outcome Evaluated	Test Used by Oral *Hp*	Test Used by Gastric *Hp*	Statistics and Interpretation
Liu et al. (2009) [15]	China	Cross-sectional study	443 (18–79)	Prevalence of gastric infection in patients with the presence of *Hp* in dental plaque.	PCR	Rapid urease test + Giesma staining/PCR/smear examination	*t*-test: *p* < 0.05; the prevalence of gastric infection was statistically higher in *Hp*+ patients in dental plaque compared to *Hp*−
Alagl et al. (2019) [10]	Saudi Arabia	Cross-sectional study	120 (>18)	To evaluate the association between gastric colonization and oral *Hp*+ association between oral colonization of *Hp* and dental diseases	PCR	Giesma staining	the presence of gastric and oral *Hp* in patients with good oral hygiene and moderate socioeconomic status is not significantly associated with periodontal disease.
Ansari et al. (2018) [16]	Pakistan	Cross-sectional study	567 (20–80)	Identify the presence of *Hp* in the oral mucosa and discover its relationship with infection and gastric complications.	PCR	PCR	Pearson’s χ; *p* > 0.05, no significant association was shown between the presence of oral *Hp* and gastric infections/complications between cases and controls. OR = 1.458, 95%CI = 0.659–3.226; oral *Hp* increases the risk of developing grade II esophageal expenditure reflux. OR = 1.215, 95%CI = 0.285–5.181; oral *Hp* increases the risk of normal upper GI mucosa with loose esophageal sphincters. OR = 2.187, 95%CI = 0.225–21.278; oral *Hp* increases the risk of duodenal ulcer/duodenitis. OR = 2052, 95%CI = 1002–4201; significantly increased risk of babA (gastritis gene).
Eskandari et al. (2010) [17]	Iran	Cross-sectional study	67 (average of 42.3)	Detect the presence of *Hp* in dental plaque and determine its association with *Hp*+ gastritis.	PCR	Rapid urease test	Chi-square and Fisher’s exact test, *p* = 0.012; significant association between the presence of *Hp* in dental plaque and gastritis.
Bürgers et al. (2008) [18]	Germany	Cross-sectional study	94 (average 53)	To evaluate the concomitant presence of *Hp* in the oral cavity and stomach + the impact of the presence of the bacteria on dental and systemic parameters.	PCR	PCR + DNA sequencing	Chi-square test (*p* = 0.05) to detect differences in prevalence.
Al-Refai et al. (2002) [10]	Saudi Arabia	Cross-sectional study	135 (21–76)	(1) To study the association between the presence of *Hp* in dental plaque and its presence in the stomach (2) To know the effect of oral hygiene on the presence of *Hp* in dental plaque and stomach. (3) To determine the correlation between the presence of *Hp* in dental plaque and periodontal condition.	Culture + rapid urease test	Urease Rapid test	Chi-square 42.629 (*p* < 0.0001); highly statistically significant association between plaque urease and gastric urease No statistically significant association was observed between plaque urease and gastric urease with oral hygiene parameters (use of toothbrush, dental floss) or with dental plaque and gingival and periodontal indices used in the study.
Al Asqah et al. (2009) [19]	Saudi Arabia	Cross-sectional study	101 (average 40.77 ± 14.15)	To evaluate whether the presence of *Hp* in dental plaque in periodontal and non-periodontal patients has a correlation with gastric pathology.	Culture + rapid urease test	Rapid urease test	Chi-square, *p* < 0.05; patients with periodontitis had a significantly higher rate of positive oral and gastric *Hp* test results compared to patients without periodontitis alone.
Silva Rossi-Aguiar et al. (2009) [20]	Brazil	Cross-sectional study	43 (average 46.9)	Assess the presence of *Hp* in oral cavity of patients with functional dyspepsia (epigastric pain syndrome).	PCR	Rapid urease test and urea breath tests.	*Hp* was found in the stomachs of 30 patients (69.7%). A total of 144 samples were collected from the oral cavity, 80 (55.5%) of which were positive for rapid urease testing. However, *Hp* DNA was not detected in any oral sample by PCR.
Silva et al. (2010) [21]	Brazil	Cross-sectional study	115 (mean 49.6 ± 5.8).	To evaluate the presence of oral *Hp* in periodontal and gastric patients. Four groups: (A) with gastric diseases and periodontal disease; (B) with gastric diseases and without periodontal disease; (C) no gastric diseases and no periodontal disease, (D) no gastric diseases and periodontal disease.	Modified Giemsa staining, hematoxylin-eosin, and PCR	PCR	Fisher’s exact test; *Hp* was detected in supragingival plaque of 9/36 (25%) of group A, 1/31 (0.3%) of group B, 0 (0%) of group C and 3/36 (8.3%) of group D. No *Hp*+ positive subgingival samples were found. A statistically higher prevalence of *Hp* resulted in groups A and D when compared to groups B and C (*p* < 0.05).
Bago et al. (2009) [22]	Croatia	Cohort Study	97 (35–74)	To evaluate the presence of *Hp* in the oral cavity of patients with *Hp*+ gastric infection.	PCR	C-urea breath test	Chi-square; of the total of 56 individuals with chronic periodontitis, Hp was detected in the oral cavity of 23 patients (41.1%). Twelve saliva samples (21.4%), 15 (26.8%) supragingival plaque samples, and 17 (30.4%) subgingival plaque samples were positive for *Hp*.

*Hp* = *Helicobacter pylori*; PCR = Polymerase chain reaction; OR = Odds ratio; DNA = Deoxyribonucleic acid.

**Table 3:** Methodological quality assessment using the JBI tool.

Study	D1	D2	D3	D4	D5	D6	D7	D8	Overall
Liu et al. (2009) [15]									Moderate quality
Alagl et al. (2019) [10]									Moderate quality
Ansari et al. (2018) [16]									Low quality
Eskandari et al. (2010) [17]									Moderate quality
Bürgers et al. (2008) [18]									Low quality
Al-Refai et al. (2002) [9]									Low quality
Al Asqah et al. (2009) [19]									Moderate quality
Silva Rossi-Aguiar et al. (2009) [20]									Low quality
Silva et al. (2010) [21]									High quality
Bago et al. (2009) [22]									Low quality

Legend: Green: low risk of bias; Red: high risk; Yellow: Unclear risk. D1: Were the criteria for inclusion of the sample clearly defined?; D2: Were the study subjects and the scenario described in detail?; D3: Was exposure measured validly and reliably?; D4: Were objective and standard criteria used for the measurement of the condition?; D5: Have confounding factors been identified?; D6: Have strategies been stated to deal with confounding factors?; D7: Have the results been measured validly and reliably?; D8: Was an appropriate statistical analysis used?
